# Identification and Characterization of a Master Transcription Factor of Th1 Cells, T-bet, Within Flounder (*Paralichthys olivaceus*)

**DOI:** 10.3389/fimmu.2021.704324

**Published:** 2021-06-28

**Authors:** Hongfei Tian, Jing Xing, Xiaoqian Tang, Heng Chi, Xiuzhen Sheng, Wenbin Zhan

**Affiliations:** ^1^ Laboratory of Pathology and Immunology of Aquatic Animals, KLMME, Ocean University of China, Qingdao, China; ^2^ Laboratory for Marine Fisheries Science and Food Production Processes, Qingdao National Laboratory for Marine Science and Technology, Qingdao, China

**Keywords:** t-box expressed in T cells, T helper type 1 cells, cytokines, differentiation, *Paralichthys olivaceus*

## Abstract

T-bet, a T-box family member, is a transcription factor essential for the differentiation of naive CD4^+^ T cells into Th1 cells that are involved in both innate and adaptive immune responses. In this study, the transcription factor T-bet of flounder (*Paralichthys olivaceus*) was cloned and characterized, and its expression profile after infection was analyzed. T-bet^+^ cells were identified in flounder, and the expression and localization of T-bet in T lymphocyte subsets and B lymphocytes were investigated. Finally, the proliferation of T-bet^+^ cells, T lymphocyte subsets, and B lymphocytes were studied after stimulation with IFN-γ, IL-2, and IL-6, respectively, and the variations of some transcription factors and cytokines in CD4^+^ T lymphocyte subsets were detected. The results showed that T-bet in flounder consists of 619 aa with a conserved T-box DNA binding domain. T-bet was abundantly expressed in the spleen, head kidney, and heart, and it was significantly upregulated after infection with *Vibrio anguillarum, Edwardsiella tarda*, and Hirame rhabdovirus, especially in the group of *Edwardsiella tarda*. A polyclonal antibody against recombinant protein of T-bet was prepared, which specifically recognized the natural T-bet molecule in flounder. T-bet^+^ cells were found to be distributed in the lymphocytes of peripheral blood, spleen, and head kidney, with the highest proportion in spleen, and the positive signals of T-bet occurred in the cell nucleus. T-bet was also detected in the sorted CD4-1^+^, CD4-2^+^, CD8^+^ T lymphocytes, and IgM^+^ B lymphocytes. In addition, T-bet^+^ cells, coordinated with CD4-1^+^ and CD4-2^+^ T lymphocytes, were proliferated after stimulation with IFN-γ, IL-2, and IL-6. Especially in sorted CD4-1^+^ and CD4-2^+^ T lymphocytes, IFN-γ and IL-2 were able to upregulate the expression of T-bet, forming a positive feedback loop in Th1-type cytokine secretion. These results suggest that T-bet may act as a master transcription factor regulating flounder CD4^+^ T lymphocytes involved in a Th1-type immune response.

## Introduction

Naive CD4^+^ T cells, on antigenic stimulation, become activated, expand, and differentiate into different effector subsets, including Th1 cells and Th2 cells, Th17 cells, regulatory T (Treg) cells, and follicular helper T (Tfh) cells (1–3). The differentiation decision is predominantly governed by antigen nature, the cytokines in the microenvironment, and the strength and duration of the interaction of the T-cell antigen receptor with antigen (4, 5). These effector cell subsets are crucial for the induction of the most appropriate immune response toward a particular pathogen. Th1 cells are characterized by their production of IFN-γ, IL-2, and involved in cellular immunity against intracellular pathogens (6). Th2 cells produce cytokines IL-4, IL-5, and IL-13 that stimulate B cells to secrete different antibody isotypes and thus control extracellular infections (7). Th17 cells produce IL-17A, IL-17F, IL-22, and play important roles in the clearance of extracellular bacteria and fungi, especially at mucosal surfaces (8). Treg cells have an essential role in regulating Th1-, Th2-, and Th17-type responses and are responsible for peripheral tolerance (9). Tfh cells are subsets of helper T cells that regulate the maturation of B cell responses (10, 11). Furthermore, Th9 and Th22, some new Th cells also have been identified and characterized in mammals (2). In brief, the complexity and plasticity of CD4^+^ T cell differentiation plays critical roles in orchestrating adaptive immune responses to a variety of infectious pathogens, allergens, and self-antigens (12).

T-box expressed in T cells (T-bet) is a member of the T-brain1 subfamily of T-box transcriptional factor genes that possess highly conserved DNA-binding domain (13). In mammals, T-bet performs a range of immunomodulatory functions in a variety of immune cell lineages. Briefly, T-bet plays a unique role in the differentiation of the helper T cells by promoting Th1 differentiation, and in the repression of Th2 and Th17 programs (14). In addition, together with another T-box family member, Eomes, T-bet controls the differentiation and maturation of CD8^+^ T cells (15). Moreover, T-bet is also expressed in B cells, dendritic cells, NK cells, and NKT cells (16). T-bet in B cells was required for IgG class switching and antibody production (17–19), and also found to be upregulated in B cells associated with aging (20). Additionally, T-bet controls the production of IFN-γ in dendritic cells (21) and suppresses the immature gene signature during murine NK cell development (22). In other words, because of the extensive and imperative functions in the immune system, T-bet has become a hot topic for immunologists.

In fish, there has been a considerable amount of work undertaken on T lymphocytes immunity over the last few decades, and a large number of related genes have been cloned, such as T-cell receptor, CD3 homologs, co-receptor, co-stimulatory or co-inhibitory molecules, cytokines, and transcription factors (23, 24). However, although we know a lot about the immune responses of T cells to different antigens, relatively little is known about the differentiation of T lymphocyte subsets, and whether Th subsets exist in fish. Fish T-bet has been cloned and characterized in several species because of its importance in the lineage determination of Th1 cells. T-bet was first cloned in ginbuna crucian carp (*C. auratus langsdorfii*), and ginbuna T-bet was strongly expressed in peripheral blood leukocytes, head kidney, spleen, and IgM^+^ lymphocytes (25). In zebrafish, T-bet and IFN-γ were up-regulated in spleen and head kidney after fish stimulated with Poly I:C (5), whereas trout T-bet was up-regulated in splenocytes by a T-cell stimulant PHA (24). In addition, the expression of T-bet in trout was inhibited by *Yersinia ruckeri* infection and strongly increased coordinating with IFN-γ and IL-2 during a parasitic infection (24). The pathogen-modulated expression of T-bet in salmon or grass carp also suggest that T-bet is involved in immune responses in teleosts (16, 26). However, little is known about the precise roles of T-bet in IFN-γ production and differentiation of CD4^+^ T cells in fish. Thus, in the present study, we have prepared the antibodies against flounder T-bet, identified T-bet^+^ cells at the cellular level, and analyzed its expression in T/B lymphocytes and the regulation of T-bet by cytokines.

## Materials and Methods

### Animals

Healthy flounders (*Paralichthys olivaceus*), of 15 to 20 cm, were obtained from a fish farm in Rizhao, Shandong province, China. The flounders were acclimated in recirculating seawater at 21°C for a week, and used for the following studies. Healthy New Zealand white rabbits (females), weighing 3 kg, were purchased from Qingdao Animal Experimental Center (Shandong, China), and they were used for antibody production. Animals were anesthetized with MS-222 prior to sampling. The animals used in the present study were carried out strictly in line with the procedures in the Guide of the Use of Experimental Animals of the Ocean University of China. The methods used in the animal experiments of this study were approved by the Instructional Animal Care and Use Committee of the Ocean University of China (permit number: 20150101). All possible efforts were dedicated to minimizing suffering.

### Sequence Analysis

The mRNA sequence of flounder T-bet was retrieved from the NCBI database with accession no. KR822591.1. The homology search of nucleotide and protein sequence of flounder T-bet were conducted with BLAST program (http://www.ncbi.nlm.gov/blast), and then multiple alignments of amino acid sequences were conducted in DNAMAN software. The structural features of protein sequence were analyzed by SMART (http://smart.embl-heidelberg.de/). Moreover, MEGA 5.0 software was used to construct and analyze a phylogenetic tree using the neighbor-joining method with 1,000 bootstrap trials.

### Expression Analysis of T-bet in Tissues

The intestine, head kidney, leukocytes, spleen, gill, skin, heart, liver, and brain were collected aseptically from six healthy flounder, respectively, and the tissues were separately used as a sample to extract RNA. The total RNA was extracted by Trizol method according to the manufacturer’s instructions. The cDNA was synthesized by using Reverse Transcriptase M-MLV kit (TaKaRa, China) according to the manufacturer’s instructions. And then, the cDNAs of the intestine, head kidney, leukocytes, spleen, gill, skin, heart, liver, and brain were used as templates in q-PCR. The specific primers were used to amplify T-bet gene fragment. Each assay was performed in triplicate using the “18s” as internal control. All the primers used in this study were listed in **Table 1**. q-PCR was carried out using SYBR Green I Master Mix (Roche, Switzerland) in a LightCycler^®^ 480 II Real-Time System (Roche, Switzerland) as previously described (27). All data were analyzed with the 2^−ΔΔCt^ method. The infection experiments were proceeded as described previously (27). *Vibrio anguillarum, Edwardsiella tarda*, and Hirame rhabdovirus virus (HIRRV) were stored in our lab. The bacterial (1.0 × 10^8^ cfu/ml) and HIRRV suspensions (10^5^ TCID_50_) were used in injection experiment. Sixty-three of the healthy fish were randomly divided into three groups, and the fish were injected in the abdominal cavity with 100 μl of *V. anguillarum*, *E. tarda*, and HIRRV, respectively. The spleen and head kidney were randomly collected at 0, 12, 24, 36, 48, 72, and 96 h from three fish in each groups. The partial of spleen and head kidney from three fish was separately used for RNA extraction. The modulation of flounder T-bet expression after infection was determined using q-PCR. Each of the samples was repeated in triplicate.

**Table 1:** Primers used in this study.

Primer name	Primer sequence (5′→3′)	Accession
**Protein Expression**		
T-bet-F	CGGGATCCCACATCGTGGAGGTGAAGG (Hind III)	KR822591.1
T-bet-R	ACGCGTCGACGTGGGCGTAATAGCCATAGT (Sal I)	
**RT-PCR**		
TCRα-F	CTGCTGCTTCTTTCAATCCTA	AB053227.1
TCRα-R	GCATCAGACCACAGAGCCACC	
TCRβ-F	GATTCCAAGCCTCAACACCTT	AB053228.1
TCRβ-R	CGCACAGATAAACAGCCTCAT	
CD3ε-F	ATGACCGGGACGATAATTCTGATGA	AB081751.1
CD3ε-R	CATAGTCAGGAGATGGGACAGGTGG	
CD4-1-F	CACCCTAAAGCCTCAAGTGGAAAT	AB643634
CD4-1-R	AAGTTTTCTGGTTGGATTTGTTTGA	
CD4-2-F	TTGGTGGGGTGATGTACACAGAG	AB640684
CD4-2R	CACAGTTGGGGCACGATGTCTC	
CD8α-F	GGTGAAACCAGTTCTATCATCCCTT	AB082957
CD8α-R	TGGTGGTGCGGGCATCTC	
CD8β-F	GTCACCCGAAGAAGAATTTTGC	AB643633
CD8β-R	ATCTTCTGAAAGTGGTGGCG	
IgM-F	GAACTGAAAGTGTCTGCCTTCTATG	AF226284
IgM-R	CCATTCTCGCTTTTATGTTCCTC	
IgD-F	TGGGGACAAGGGACAAAGG	AB052658
IgD-R	GCGAGGCAGCCAAGAGTG	
CD79β-F	GCAGCATCAGAATAGCGACA	KC345763.1
CD79β-R	TGTTCCTCAAAGCCACCTCTGCC	
T-bet-F	GTTCATCGCTGTCACTGCCTATC	KR822591.1
T-bet-R	ACCCTCCTCTTCTTGTTGTCCC	
β-actin-F	GATGGTGGGTATGGGCCAGAAG	HQ386788.1
β-actin-R	ATGTCACGCACGATTTCCCTCTC	
**qPCR**		
T-bet-F	GCCGACATCAGCAGTCACCT	KR822591.1
T-bet-R	TGTGCGTAAAACCTGCCG	
IFN-γ-F	TGTCAGGTCAGAGGATCACACAT	AB435093
IFN-γ-R	GCAGGAGGTTCTGGATGGTTT	
IL-2-F	ATGGAGCACTTTATTGGGATT	KY307833
IL-2-R	TCACATTTGTTGGAGCGTAGA	
GATA3-F	CAGGAGGACAAAGAGTGCATAAAGT	XM_020108979.1
GATA3-R	GAAGATGACCCACCTATCAGGCTAC	
IL-10-F	TACGAAGCGAACGATGACCTA	XM_020086558.1
IL-10-R	GCTCGTCGAAGATTTGCTGTAT	
IL-17A-F	CCTGGATGTGACTCCTTGTTGG	XM_020111881.1
IL-17A-R	GACGCTCTGGTAGATGGGAACT	
IL-6-F	GTAACCGCTCACCACCAGAAAGA	DQ267937.1
IL-6-R	TCTGGCGTGCAAGAGGATGGAGC	
TNF-α-F	GTCCTGGCGTTTTCTTGGTA	AB040448
TNF-α-R	CTTGGCTCTGCTGCTGATTT	
18sRNA-F	GGTCTGTGATGCCCTTAGATGTC	EF126037
18sRNA-R	AGTGGGGTTCAGCGGGTTAC	

### Production of Polyclonal Antibodies Against Flounder T-bet

The cDNA sequence encoding the C-terminus of flounder T-bet (990bp, encoding 330aa) was amplified. The PCR products were purified and cloned into pEASY-T1 vector (TAKARA, Japan). After being sequenced, the PCR products were cloned into pET-28a vectors and verified to be inserted successfully. Then, the recombinant pET-28a-T-bet vectors were transformed into *Escherichia coli* Transetta (DE3) (TAKARA, Japan) and induced with isopropy-β-d-thiogalactoside (IPTG) for 4 h at 30°C during exponential growth. Finally, the recombinant proteins T-bet (rT-bet) were affinity-purified using His Trap™ HP Ni-Agarose (GE healthcare China, Beijing, China) according to the instructions. The induced recombinant *Escherichia coli* lysates and purified rT-bet were detected by sodium dodecyl sulfate polyacrylamide gel electrophoresis (SDS-PAGE) and stained with Coomassie brilliant blue R-250.

The concentrations of rT-bet were determined by the Bradford method and rT-bet were used for immunization of New Zealand white rabbits according to a previous method (28). After three boosters, the serum samples were collected and purified by protein G agarose affinity chromatography (Pierce/Thermo Scientific), and then the rabbit anti-flounder rT-bet polyclonal antibodies (Abs) were obtained. The Abs titer was tested by immunosorbent assay (ELISA), and the specificity of the Abs was analyzed using Western blotting and mass spectrometry analysis. The anti-flounder rT-bet Abs was diluted 1:1000 in PBS and used in following experiments.

### Preparation of Lymphocytes and Western Blotting Analysis

The lymphocytes in peripheral blood, spleen, and head kidney were isolated from flounder according to a method described previously (29). In brief, the peripheral blood was drawn from the caudal vein and diluted in solution (65% RPMI-1640 containing 20 IU mL^−1^ heparin, 0.1% w/v NaN_3,_ and 1% w/v BSA). The head kidney and spleen were extirpated, and cell suspensions were prepared by squeezing the tissue with 65% RPMI-1640 solution through a nylon gauze filter. Then the peripheral blood and cell suspensions were centrifuged, and the supernatants were laid over a 1.020- to 1.070-g/cm^3^ discontinuous Percoll density gradient. After centrifugation, the lymphocytes layers from the Percoll interface were collected. Whole protein extracts were extracted from the lymphocytes (1×10^7^ cells/ml) of peripheral blood, spleen, and head kidney, and then used for Western blotting. The recombinant proteins T-bet and lymphocytes lysates from flounder tissues went through SDS-PAGE and transferred onto PVDF membrane (Merck Millipore, Darmstadt, Germany). Then the PVDF membranes were blocked with 3% BSA and then incubated with Abs as primary antibody for 1 h at 37°C, and the serum of unimmunized rabbit was used as a negative control. Goat-anti-rabbit Ig-alkaline phosphatase conjugate (Sigma, USA) diluted with PBS (1:3000) according to instructions was used as secondary antibody, the membranes were incubated with secondary antibody for 1 h at 37°C. After the last washing, the bands were detected with fresh substrate solution (100 mM NaCl, 5 mM MgCl_2_, and 100 mM Tris, pH 9.5) containing nitroblue tetrazolium (NBT; Sigma, St. Louis, MO, USA), and 5-bromo-4-chloro-3-indolyphosphate (BCIP; Sigma, St. Louis, Mo, USA) for about 5 min. Then the immune-reactive bands to lymphocytes in gels were excised for mass spectrometry analysis.

### Immunofluorescence and Flow Cytometry

The lymphocytes (1 × 10^7^ cells/ml) isolated from peripheral blood, spleen, and head kidney were fixed and permeabilized, and then incubated with the Abs for 1 h at 37°C. After washing three times with PBS, the lymphocytes were resuspended and incubated with Alexa Fluor 647-conjugated goat anti-rabbit IgG (1:1000 diluted in PBS, Sigma) for 45 min at 37°C in the dark. After washing as above and resuspended in PBS, the lymphocytes suspensions were analyzed using Accuri C6 flow cytometer (BD, USA). For immunofluorescence observation in microscope, the cells were counterstained with DAPI (Bio-Legend) for 10 min at 37°C in dark. After the last washing, 20-μl lymphocytes suspension was dripped onto APES coated slides, and the cells were settled and fixed onto slides after 2 h, and then observed under a fluorescence microscope (Olympus DP70, Tokyo, Japan). Unimmunized rabbit serum was used as negative controls instead of Abs as primary antibody.

### Cell Sorting and RT-PCR

The lymphocytes isolated from head kidney of flounder were incubated with the monoclonal antibodies against CD4-1, CD4-2, IgM or mouse polyclonal antibodies anti-CD8β, respectively (29–31). After the lymphocytes were incubated with the secondary antibody (Alexa Fluor 488-conjugated goat anti-mouse IgG, 1:1000 diluted in PBS), CD4-1^+^, CD4-2^+^, CD8^+^ T lymphocytes, and IgM^+^ B lymphocytes were isolated by flow cytometry (BD FACSAria). The purity of harvested cells was analyzed after being sorted (≥98%), then the sorted cells were processed for RNA extraction. The expression of T-bet in flounder T lymphocyte subsets and B lymphocytes was determined by RT-PCR.

### Double Immunofluorescence Staining

The lymphocytes were extracted from the head kidney of flounder, adjusted to a concentration of 0.5 × 10^7^ cells/ml, settled on adherent slides for 2 h, fixed in 4% paraformaldehyde, and then frozen at −20°C for the next double immunofluorescence staining. The lymphocytes were permeabilized by 0.1% TritonX-100, and then incubated with the primary antibody and secondary antibodies. The mixture of two kinds of antibodies (rabbit anti-T-bet polyclonal antibodies have mixed with mouse anti-IgM, anti-CD4-1, anti-CD4-2 monoclonal antibodies, and mouse anti-CD8 polyclonal antibodies, respectively) was used as the primary antibody, and the mixture of Alexa Fluor 488-conjugated goat-anti-mouse IgG and 647-conjugated goat anti-rabbit IgG was used as the secondary antibody. After the cells were counterstained with DAPI, the cells were then observed under a fluorescence microscope (Olympus DP70, Tokyo, Japan). The mixture of unimmunized rabbit and mouse serum was used as a negative control.

### Stimulation of Lymphocytes by Cytokines

Lymphocytes were isolated from 18 flounder head kidney with aseptic technique in each group, and 72 (a half of fish used for flow cytometry, and a half of fish used for cell sorting) flounder were totally used in this experiment. Then, the cells were transferred to 24-well culture plates (800 μl/well) to ensure 5 × 10^6^ cells per well, and cultured in L-15 medium with 10% fetal calf serum and penicillin/streptomycin supplemented with IFN-γ, IL-2, and IL-6 (100 ng/well) (32). PBS was added to the cells as control. The lymphocytes were collected after stimulation at 0, 1, and 3 days, then the variations of T-bet^+^ cells, CD4-1^+^, CD4-2^+^, CD8^+^ T lymphocytes, and IgM^+^ B lymphocytes were analyzed by flow cytometry. Furthermore, CD4-1^+^ and CD4-2^+^ T cells were isolated after stimulation, the cytokines and transcript factors in CD4^+^ T cells were detected by q-PCR.

### Statistical Analysis

The experiments in this study were repeated in triplicate, and the data were expressed as mean ± SD. *p* Values less than 0.05 was considered significant. One-way analysis of variance (ANOVA) and Duncan’s multiple comparisons were performed by using Statistical Product and Service Solution (SPSS) software (Version 20.0; SPSS, IBM, BY, USA).

## Results

### Sequence Analysis of Flounder T-bet

**Figure 1:**
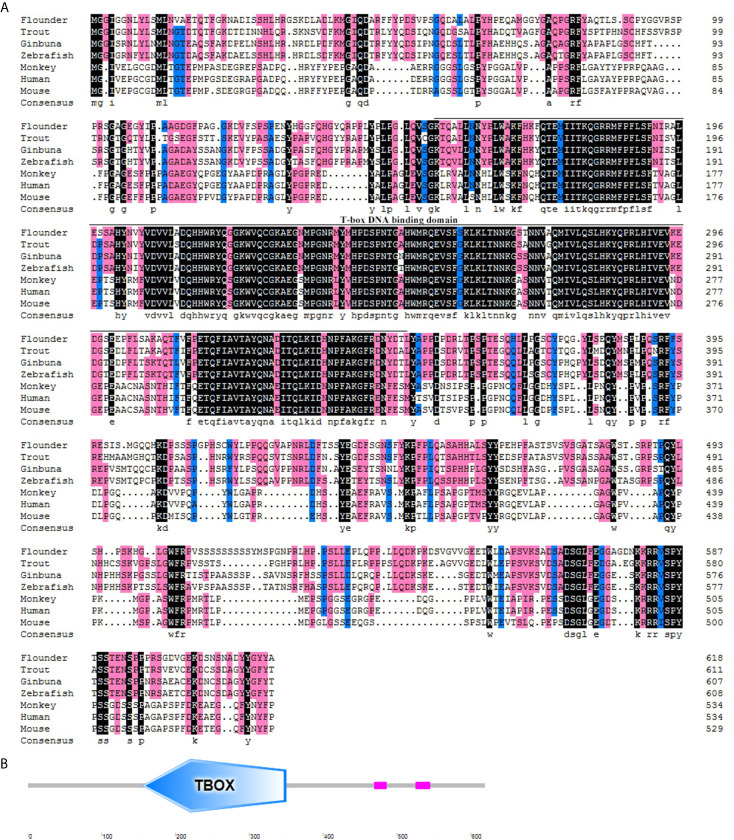
Multiple alignment and structure profile of T-bet molecules in flounder. **(A)** Multiple alignment of T-bet molecules from flounder and other vertebrates. The T-box DNA binding domain is indicated by solid line below the alignment. The accession numbers for sequences used in this analysis are given in the **Figure 2** legend. **(B)** Structure profile of flounder T-bet molecule predicted by SMART. TBOX means the T-box DNA binding domain of flounder T-bet.

**Figure 2:**
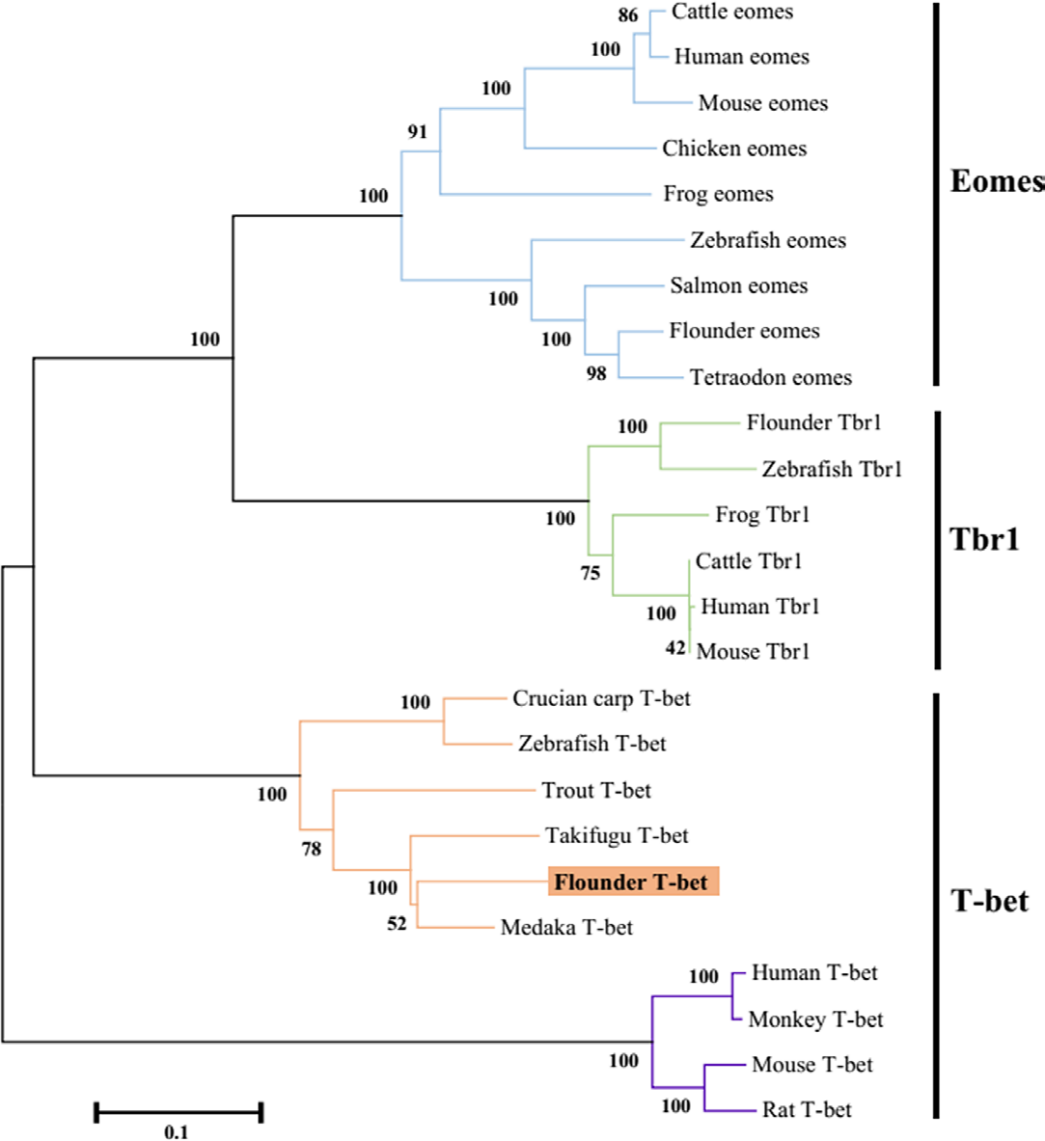
Phylogenetic tree analysis of the flounder T-bet molecule with known vertebrate members of the Tbr1 subfamily of the T-box protein family. The tree was constructed using the neighbor-joining method within the MEGA5 program. Node values represented the percent of bootstrap confidence derived from 1000 replicates. Accession numbers or ENSEMBL gene IDs are as follows: Cattle eomes, NP_001178117.1; Human eomes, O95936.3; Mouse eomes, O54839.3; Chicken eomes, XP_426003.3; Frog eomes, P79944.1; Zebrafish eomes, Q9DDU3; Salmon eomes, ACB87011.1; Flounder eomes, XP_019958075.1; Tetraodon eomes, Q4RL68; Flounder Tbr1, XP_019945371.1; Zebrafish Tbr1, NP_001108562.1; Frog Tbr1, XP_012826012.1; Cattle Tbr1, NP_001178978.1; Human Tbr1, Q16650.1; Mouse Tbr1, Q64336.2; Crucian carp T-bet, BAF73805.1; Zebrafish T-bet, NP_001164070.1; Trout T-bet, CAR95098.1; Takifugu T-bet, XP_003961133.1; Flounder T-bet, ALK27170.1; Medaka T-bet, XP_004080691.1; Human T-bet, NP_037483.1; Monkey T-bet, XP_001082367.1; Mouse T-bet, NP_062380.2; Rat T-bet, NP_001100513.1.

The flounder (*P. olivaceus*) T-bet cDNA sequence (Genbank accession no. KR822591.1) consisted of 2729 bp in length including a 271 bp 5’-UTR, a 1860 bp coding region, and a 598-bp 3′-UTR (**Figure 1A**). The flounder T-bet was a predicted 619 amino acid protein with a 196 amino acid T-box DNA binding domain (155–350 aa) (**Figure 1B**). The molecular weight of flounder T-bet was calculated 68.03 kDa. A multiple alignment of T-bet amino acid sequence in flounder and other vertebrates was depicted. Flounder T-bet shared 73.64%, 69.86%, 70.22%, 44.63%, 44.33%, and 43.14% amino acid identity with trout, ginbuna, zebrafish, monkey, human, and mouse T-bet, respectively. Although the middle T-box DNA binding domain is highly conserved among all the T-bet proteins from fish to mammals, the N-terminal and the C-terminal sequences between fish and mammals are quite divergent. Moreover, as shown in phylogenetic tree, the flounder T-bet molecule was clustered in teleost but grouped with mammalian T-bet molecules (**Figure 2**).

### Gene Expression of T-bet After Infection

**Figure 3:**
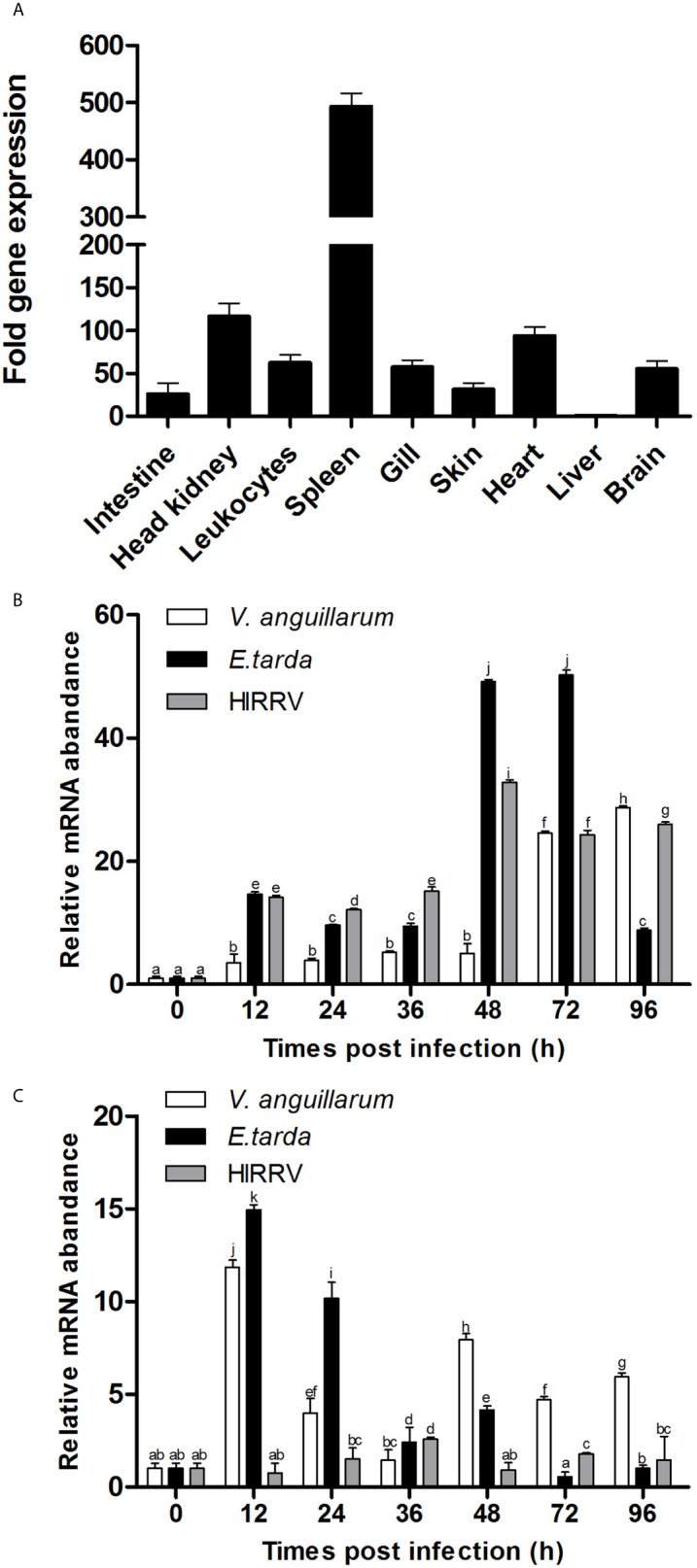
Tissue distribution of T-bet expression in healthy or infected flounder. **(A)** The expression of T-bet in healthy flounder (n=6) tissues was determined by qPCR. Gene expression data were normalized to “18s” expression using liver as a calibrator. **(B)** Variation of T-bet in spleen of flounder after infection with *Vibrio anguillarum*, *Edwardsiella tarda*, and Hirame novirhabdovirus. **(C)** Variation of T-bet in head kidney of flounder after infection with *Vibrio anguillarum*, *Edwardsiella tarda* and Hirame novirhabdovirus. The mRNA level of T-bet was normalized to that of 18S, n=3, and the mRNA level of the control fish was set as 1. All values are shown as means ± SD. Different letters (such as a, b, c, et al.) on the bar represent statistical significance (*p* < 0.05).

The T-bet mRNA expression was analyzed in healthy flounder tissues by q-PCR, and the results showed that T-bet expression levels was the highest in spleen and higher in head kidney, heart, peripheral blood leukocytes, brain, and gill, followed by skin, intestine, and liver (**Figure 3A**). To assess the involvement of T-bet in the pathology of *V. anguillarum*, *E. tarda*, and HIRRV infection, its expression was examined in the spleen and head kidney during the first few days post-challenge. In the spleen, during the healthy flounder infection with three of the pathogens, T-bet was significantly up-regulated in all the groups (**Figure 3B**). Especially after the infection of *E. tarda* and HIRRV, early and strong T-bet expressions were detected, and peaked at 48 and 72 h, the peak value of T-bet expression in the group of *E. tarda* was higher than that in the group of HIRRV. In the group of *V. anguillarum*, the maximum expression level of T-bet was at 96 h, and lower than that in the groups of *E. tarda* and HIRRV. Different from the spleen, T-bet expression in the head kidney significantly upregulated after infection of *V. anguillarum* and *E. tarda* with a peak at 12 h post challenge (**Figure 3C**), whereas a moderate T-bet expression was observed after challenge with HIRRV.

### Characterization of the Antibodies Against T-bet

**Figure 4:**
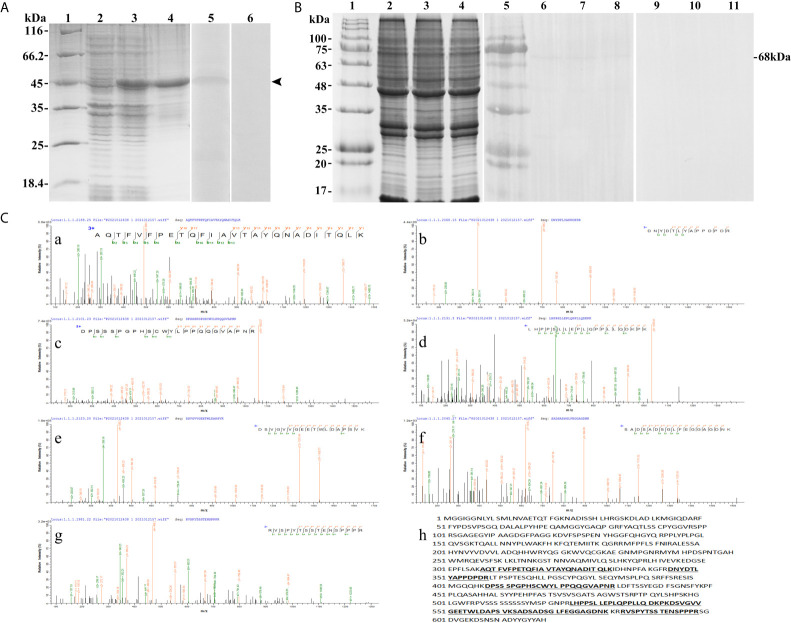
The specificity of antibodies against flounder T-bet detected by Western-blotting and Mass spectrographic analysis. **(A)** SDS-PAGE results of flounder T-bet recombinant proteins and western blotting results of antibodies to recombinant proteins. Lane 1, molecular mass marker; Lane 2, transformated *E. coli* without IPTG induction; Lane 3, transformated *E. coli* induced with IPTG; Lane 4, purified recombinant protein of founder T-bet; Lane5, Antibodies react to purified recombinant protein of founder T-bet; Lane 6, purified recombinant protein of T-bet incubated with the serum from no immunized rabbit as a negative control. **(B)** Western blotting results of antibodies to lymphocyte lysates. Lane 1and 5, molecular mass marker; Lane 2-4, SDS-PAGE results of lymphocyte lysates in peripheral blood, spleen, and head kidney, respectively; Lane 6-8, lymphocyte lysates of peripheral blood, spleen, and head kidney incubated with the antibodies of T-bet; Lane 9-11, negative control. **(C)** Mass spectrographic analysis of the immunoreactive band at 68 kDa. a-g, the LC-MS/MS results of the seven peptides in T-bet sequence. h, The matched seven peptides are underlined and bold in the sequence of T-bet.

The SDS-PAGE analysis showed that the recombinant protein of T-bet was successfully expressed in *Escherichia coli* Transetta (DE3) with pET-28a vector after IPTG induction. The molecular weight of the expressed protein was approximately 45 kDa (**Figure 4A**, lane 3), which was in accordance with predicted molecular mass. The recombinant protein of T-bet with high purity was obtained (**Figure 4A**, lane 4) and used for immunization. In addition, rabbit against T-bet Abs were successfully produced and purified, and the titer of anti T-bet Abs was 1:640,000 detected by an enzyme-linked immunosorbent assay (ELISA). The specificity of rabbit anti T-bet Abs was analyzed by Western blotting, and the results showed that the Abs could specifically recognize recombinant protein of T-bet (**Figure 4A**, lane 5). Moreover, the Abs could specifically recognize about 68 kDa bands in lymphocytes lysates of flounder peripheral blood, spleen, and head kidney, which were in accordance with the molecular mass of full T-bet protein calculated on the predicted protein sequence (**Figure 4B**, lanes 6–8). The mass spectrometry results showed that the 68-kDa protein was identified as a native flounder T-bet molecule, in which seven peptides were matched to the chain of flounder T-bet with 38% coverage of the amino acid sequence (**Figure 4C**). The results indicated the rabbit anti T-bet Abs specifically recognized flounder T-bet molecules and could be used in the following experiments.

### Identification of T-bet^+^ Cells

**Figure 5:**
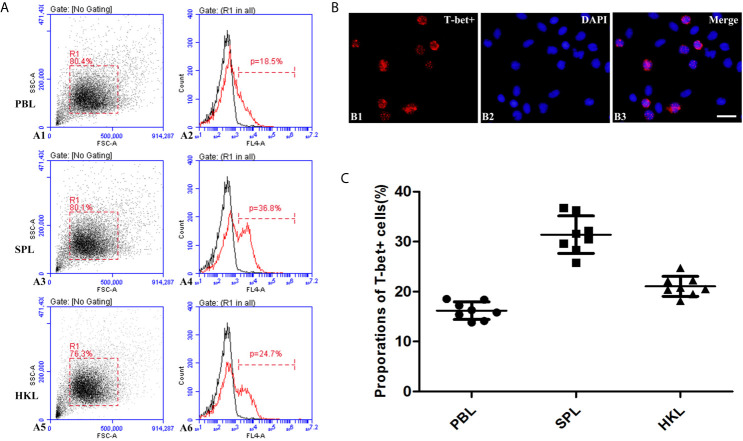
Identification of T-bet^+^ cells in flounder. **(A)** Flow cytometry results of T-bet^+^ cells in the lymphocytes of peripheral blood, spleen, and head kidney, respectively. **(B)** Immunofluorescence staining results of T-bet^+^ cells, Bar = 10µm. B1, T-bet^+^; B2, DAPI; B3, Merge. **(C)** Percentages of T-bet^+^ cells in the lymphocytes of peripheral blood, spleen, and head kidney, respectively, n = 8.

The presence of T-bet^+^ cells was detected in the extracted peripheral blood, spleen, and head kidney lymphocytes by incubation with polyclonal antibodies against flounder T-bet. Flow cytometry results showed that the percentages of T-bet^+^ cells were 16.19% ± 1.78% in peripheral blood lymphocytes, 31.4 ± 3.75% in spleen lymphocytes, 21.05% ± 2.00% in head kidney lymphocytes (**Figures 5A, C** **)**. T-bet^+^ cells were observed to be red using fluorescence microscope in splenic lymphocytes by indirect immunofluorescence, and the positive signals were mostly concentrated on the nucleus (**Figure 5B**).

### The Expression of T-bet in T and B Lymphocytes

**Figure 6:**
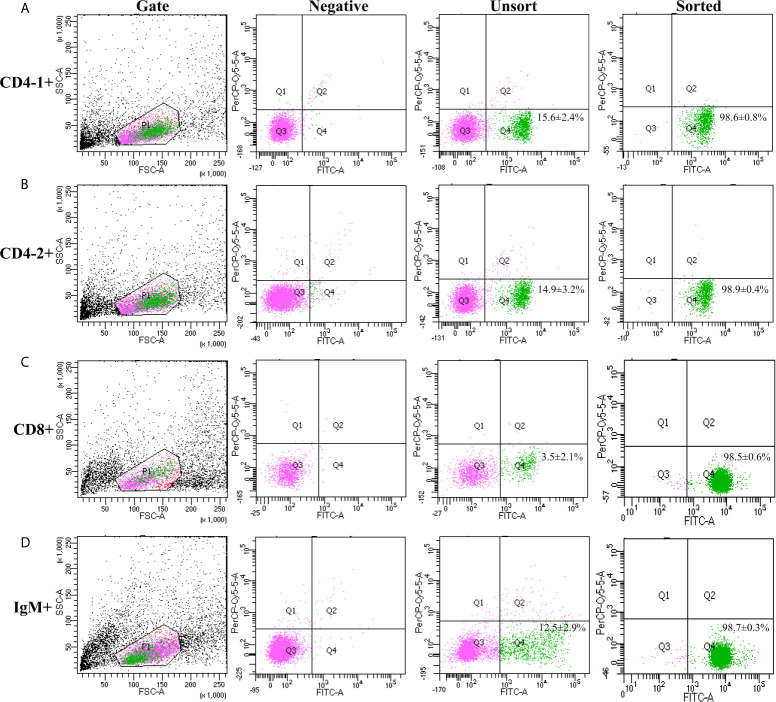
Cell sorting of CD4-1^+^, CD4-2^+^, CD8β^+^ T lymphocytes, and IgM^+^ B lymphocytes. Lymphocytes of head kidney were stained with the antibodies against CD4-1, CD4-2, CD8β, and IgM, respectively. Then the stained CD4-1^+^ **(A)**, CD4-2^+^ **(B)**, CD8β^+^ **(C)** T lymphocytes, and IgM^+^ B lymphocytes **(D)** were isolated by flow cytometry (BD FACSAria).

**Figure 7:**
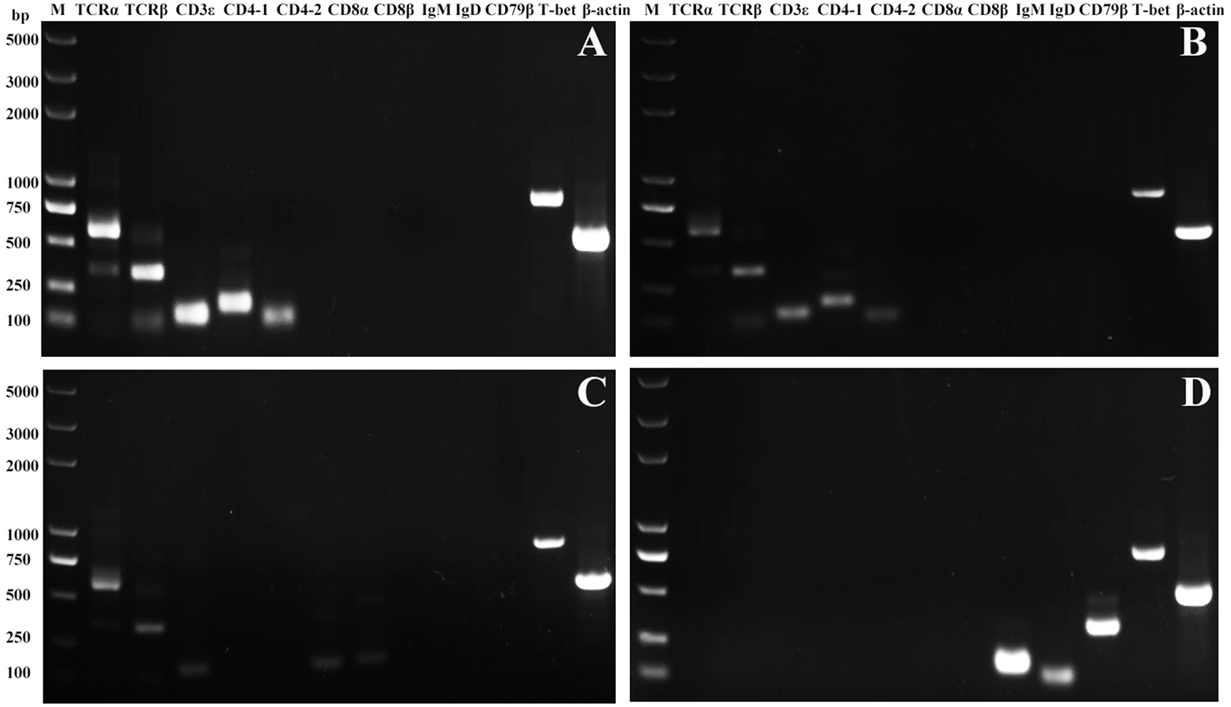
Expression of T-bet in sorted T and B lymphocytes. The total RNA of sorted CD4-1^+^, CD4-2^+^, CD8β^+^ T lymphocytes, and IgM^+^ B lymphocytes were prepared and used for RT-PCR. The surface markers of T/B lymphocytes and T-bet were detected from the sorted CD4-1^+^ **(A)**, CD4-2^+^ **(B)**, CD8^+^ **(C)** T lymphocytes, and IgM^+^ B lymphocytes **(D)**. β-actin was used as an internal control.

**Figure 8:**
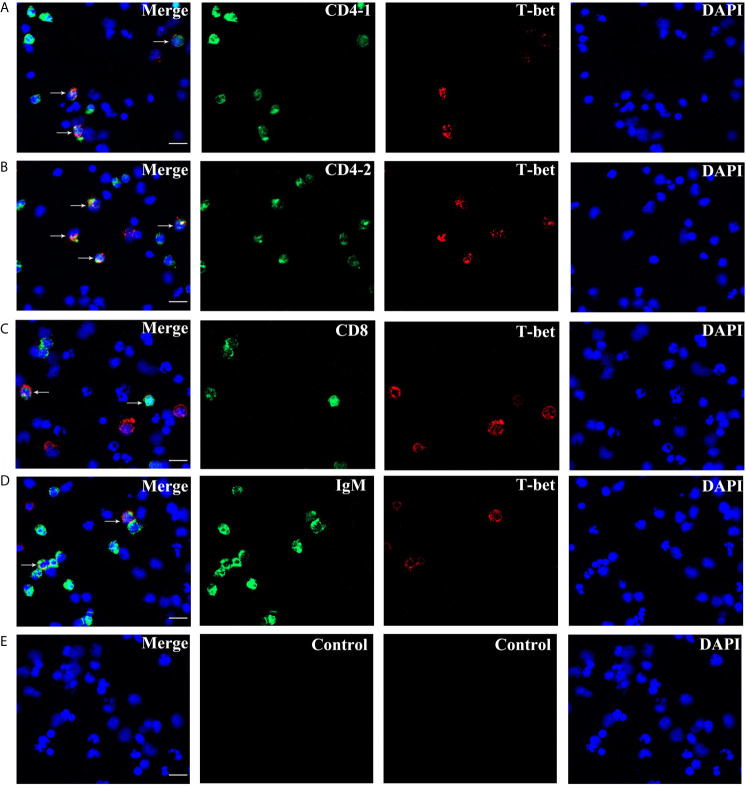
Double immunofluorescence staining results of T-bet^+^/CD4-1^+^, T-bet^+^/CD4-2^+^, T-bet^+^/CD8β^+^, and T-bet^+^/IgM^+^ cells. **(A)** T-bet^+^/CD4-1^+^ cells. **(B)** T-bet^+^/CD4-2^+^ cells. **(C)** T-bet^+^/CD8β^+^ cells. **(D)** T-bet^+^/IgM^+^ cells. **(E)** Negative controls. Rabbit anti-flounder T-bet Abs, mouse anti-flounder CD4-1, CD4-2, and IgM mAbs, and mouse anti-flounder CD8 Abs were used in this experiment. White arrows indicate double positive cells. Bar = 10 µm.

High purity of CD4-1^+^ T cells, CD4-2^+^ T cells, CD8^+^ T cells, and IgM^+^ B cells was successfully sorted from head kidney lymphocytes using the previously developed monoclonal and polyclonal antibodies (29–31). Before sorting, T lymphocyte subsets and B lymphocytes contained different proportions in the head kidney lymphocytes, and the purity of the sorted cells could be 98.5% or more as seen in the flow cytometry results after sorting (**Figure 6**). RNA were extracted from the sorted cells and RT-PCR was performed, and it was found that TCR, CD3, CD4-1, CD4-2, and T-bet genes were expressed in the sorted CD4-1^+^ T cells, and CD8, IgM, IgD, and CD79β genes were not detected. Similarly, in CD4-2^+^ T cells, the same expression of T-bet was found. Sorted CD8^+^ T cells did not express CD4 molecules, but also expressed T-bet genes. In contrast, IgM^+^ B cells did not express T cell-associated genes and expressed IgM, IgD, CD79β, and T-bet genes (**Figure 7**). These results suggest that T-bet is expressed in both T/B lymphocytes. The results of indirect immunofluorescence staining illustrated the same results. Two-color immunofluorescence staining in head kidney lymphocytes revealed the presence of T-bet^+^/CD4-1^+^, T-bet^+^/CD4-2^+^, T-bet^+^/CD8^+^, and T-bet^+^/IgM^+^ cells, and T-bet was found to be localized in the nucleus of T/B lymphocytes (**Figure 8**).

### Variations of T-bet^+^ Cell, T and B Lymphocytes After Cytokines Stimulation

**Figure 9:**
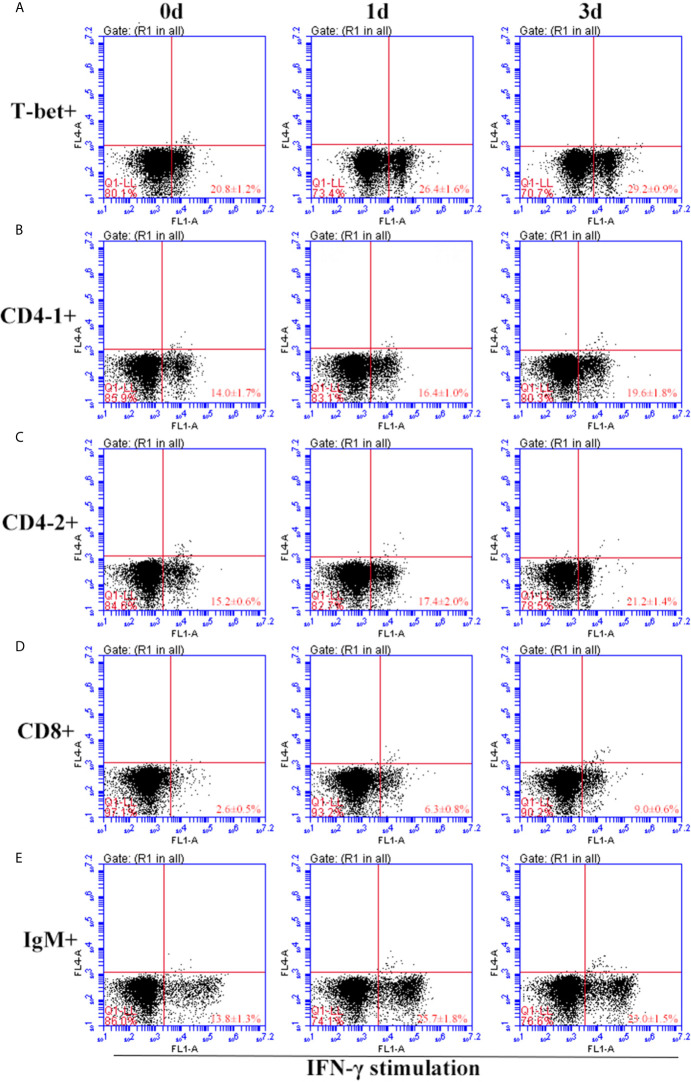
Variations of T-bet^+^ cells, CD4-1^+^, CD4-2^+^, CD8^+^ T lymphocytes, and IgM^+^ B lymphocytes after stimulation with IFN-γ. The lymphocytes were isolated from head kidney of flounder, and cultured in L-15 medium with 10% fetal calf serum and penicillin/streptomycin supplemented with IFN-γ (100 ng/well). Then the cells were collected at 0d, 1d, and 3d, the proportion of T-bet^+^ cells **(A)**, CD4-1^+^ **(B)**, CD4-2^+^ **(C)**, CD8^+^ **(D)** T lymphocytes, and IgM^+^ B lymphocytes **(E)** were detected by flow cytometry, respectively. Each figure is representative of three analyses.

**Figure 10:**
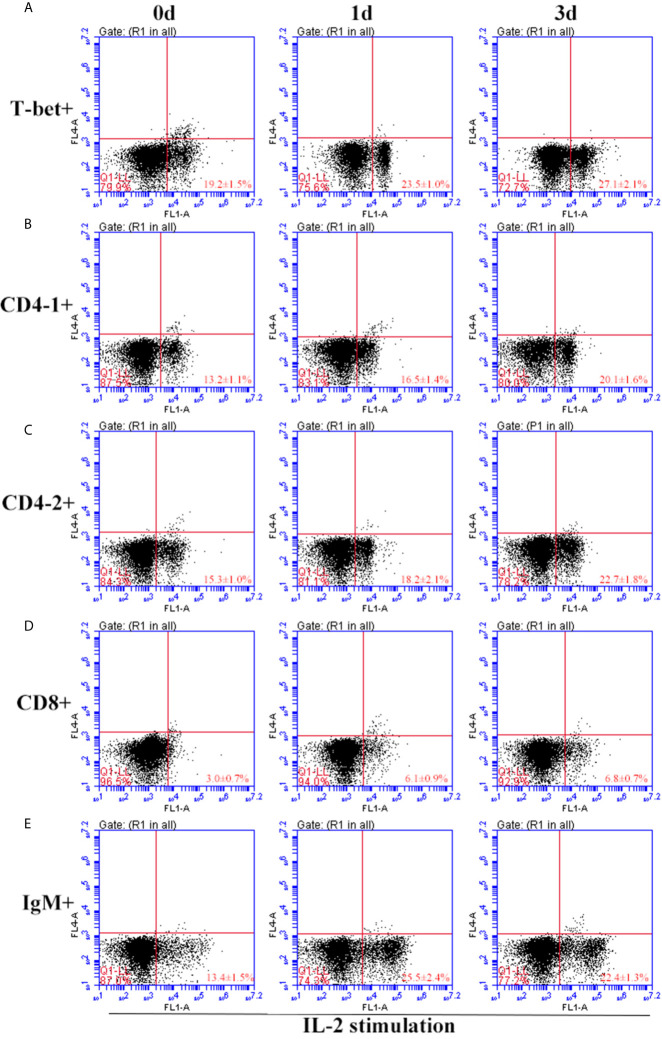
Variations of T-bet^+^ cells, CD4-1^+^, CD4-2^+^, CD8^+^ T lymphocytes, and IgM^+^ B lymphocytes after stimulation with IL-2. The lymphocytes were isolated from head kidney of flounder, and cultured in L-15 medium with 10% fetal calf serum and penicillin/streptomycin supplemented with IL-2 (100 ng/well). Then the cells were collected at 0d, 1d, and 3d, the proportion of T-bet^+^ cells **(A)**, CD4-1^+^ **(B)**, CD4-2^+^ **(C)**, CD8^+^ **(D)** T lymphocytes, and IgM^+^ B lymphocytes **(E)** were detected by flow cytometry, respectively. Each figure is representative of three analyses.

**Figure 11:**
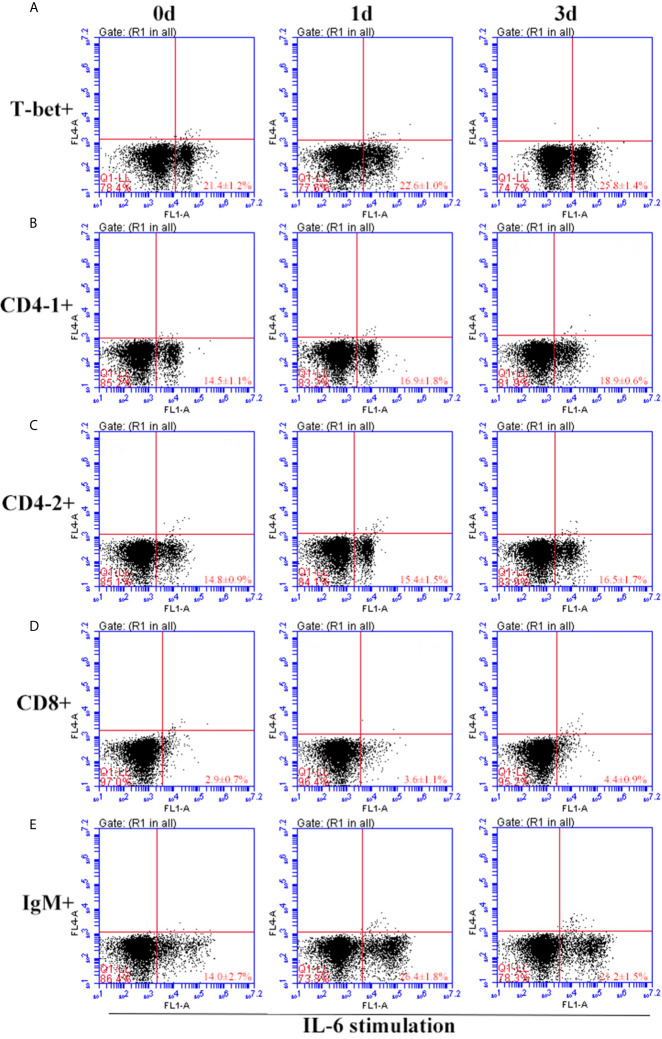
Variations of T-bet^+^ cells, CD4-1^+^, CD4-2^+^, CD8^+^ T lymphocytes, and IgM^+^ B lymphocytes after stimulation with IL-6. The lymphocytes were isolated from head kidney of flounder, and cultured in L-15 medium with 10% fetal calf serum and penicillin/streptomycin supplemented with IL-6 (100 ng/well). Then the cells were collected at 0d, 1d, and 3d, the proportion of T-bet^+^ cells **(A)**, CD4-1^+^ **(B)**, CD4-2^+^ **(C)**, CD8^+^ **(D)** T lymphocytes, and IgM^+^ B lymphocytes **(E)** were detected by flow cytometry, respectively. Each figure is representative of three analyses.

After IFN-γ, IL-2, and IL-6 stimulation, the variations of T-bet^+^ cells, CD4-1^+^, CD4-2^+^, and CD8β^+^ T cells and IgM^+^ B cells in lymphocytes of head kidney were analyzed. Head kidney lymphocytes showed varying degrees of proliferation of T-bet^+^ cells, CD4-1^+^, CD4-2^+^, and CD8β^+^ T cells and IgM^+^ B cells after the addition of recombinant cytokines stimulation. In the IFN-γ group, the proportion of T-bet^+^ cells increased significantly by day 3, up to 29.2 ± 0.9%, and CD4-1^+^, CD4-2^+^, and CD8β^+^ T cells also proliferated, reaching a peak of proliferation by day 3. In contrast, IgM^+^ B cells reached a proliferation peak of 25.7 ± 1.8% on day 1 (**Figure 9**). After IL-2 stimulation, T-bet^+^ cells in lymphocytes in the head kidney also appeared to proliferate, reaching a peak point of 27.1 ± 2.1% on day 3. Similar to the IFN-γ group, the proportion of T lymphocyte subpopulations all reached a peak on day 3, whereas the proportion of IgM^+^ B lymphocytes reached a peak on day 1 (**Figure 10**). After stimulation of head kidney lymphocytes with IL-6, T-bet^+^ cells, CD4-1^+^, CD4-2^+^, and CD8β^+^ T cells and IgM^+^ B cells proliferated less intensely than in the IFN-γ and IL-2 groups, but their percentages also appeared to increase to some extent (**Figure 11**).

### Expression of Transcription Factors and Cytokines in CD4^+^ Cells After Stimulation

**Figure 12:**
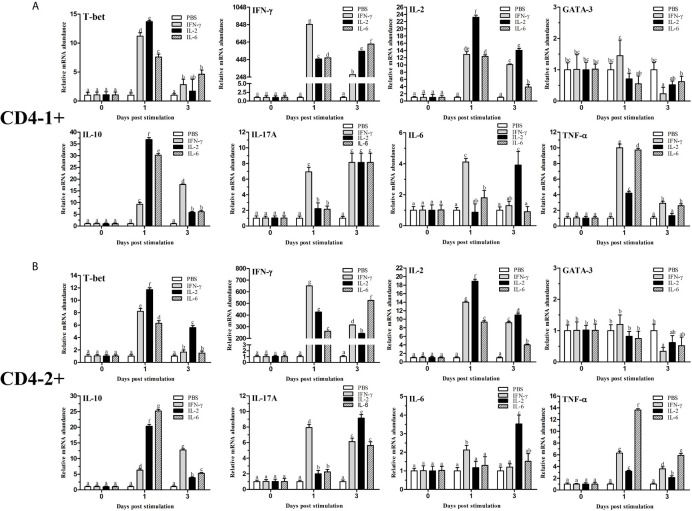
Analysis of the expression of transcription factors and cytokines in CD4-1^+^ **(A)** and CD4-2^+^ **(B)** T lymphocytes after stimulation with IFN-γ, IL-2 or IL-6. The mRNA level of each gene was normalized to that of 18s rRNA. For each gene, the mRNA level of the control fish was set as 1. The results are expressed as the mean ± SD. Different letters (such as a, b, c, et al.) on the bar represent statistical significance (*p* < 0.05).

**Figure 13:**
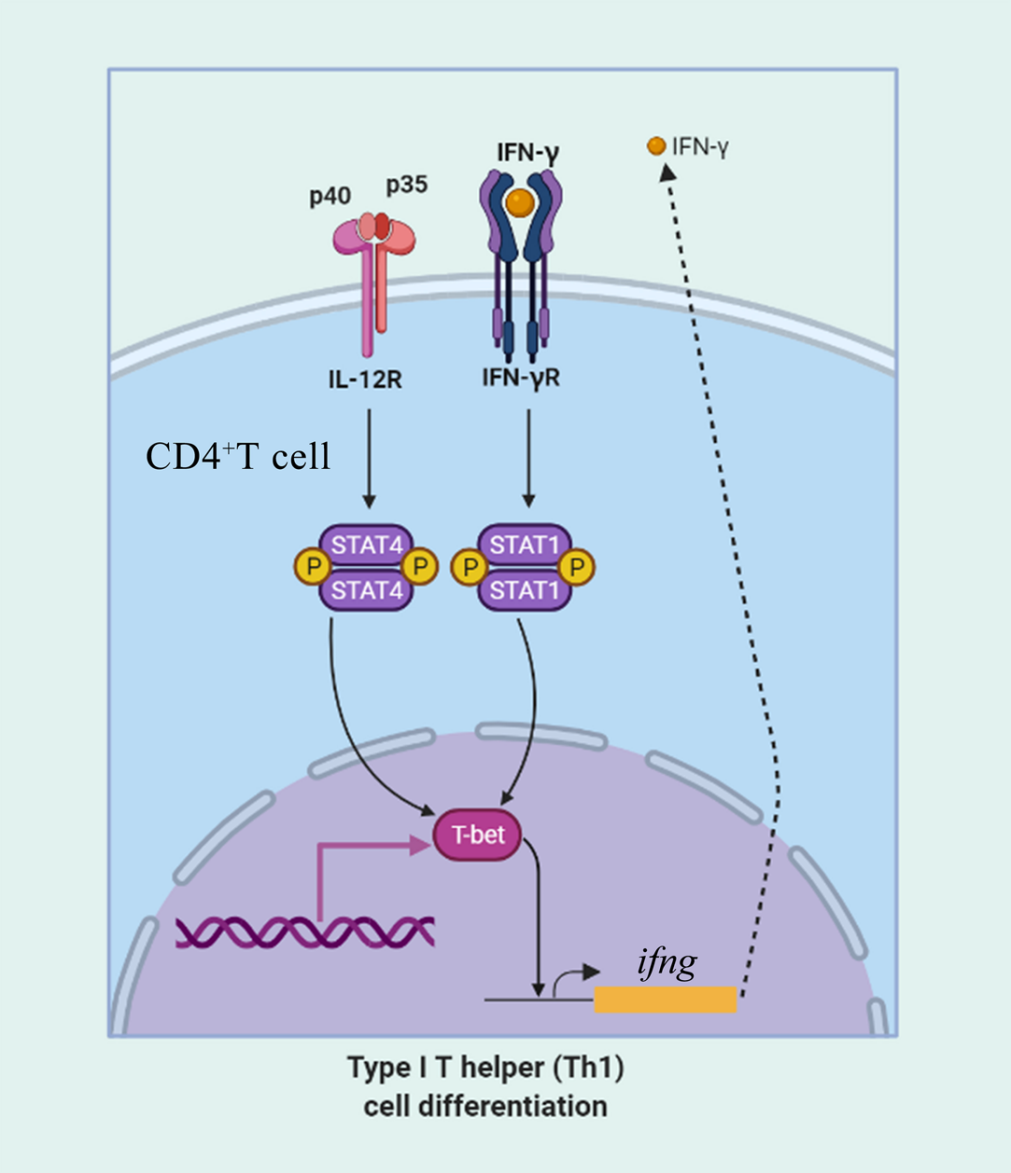
The role of T-bet in IFN-γ production and in CD4^+^ Th1 cells differentiation. CD4^+^ T cells upregulate T-bet in response to IFN-γ in a signal transducer and activator of transcription 1 (STAT1) manner leading to upregulation of IFN-γ production. IL-12 acts through STAT4 to upregulate T-bet and promote IFN-γ secretion.

After stimulation with IFN-γ, IL-2, and IL-6, CD4^+^ T cells were sorted from the lymphocytes, some transcription factors of T-helper (Th) cells and several cytokine gene expression profiles in flounder CD4-1^+^ and CD4-2^+^ lymphocytes were analyzed, the results are shown in **Figure 12**. The role of T-bet in IFN-γ production and in CD4^+^ Th1 cells differentiation was summarized in **Figure 13**. The transcription factors and cytokines showed similar expression trends in CD4-1^+^ and CD4-2^+^ T cells. The expression of T-bet was upregulated after IFN-γ, IL-2, and IL-6 stimulation, peaking at 1 day, with the most pronounced upregulation after IL-2 stimulation and decreasing thereafter. IFN-γ was also upregulated after the addition of three recombinant cytokines, especially in the group of IFN-γ, the IFN-γ gene was upregulated up to 800-fold in CD4-1^+^ T cells and 600-fold in CD4-2^+^ T cells at 1 day. In the IL-2 group, IFN-γ expression was also upregulated by 400-fold, with the difference that the peak point for CD4-1^+^ was on day 3, whereas the peak point for CD4-2^+^ was on day 1. The expression of IFN-γ was also up-regulated 500-fold in the group with the addition of IL-6, and peaked on day 3. IL-2 expression was most significantly upregulated after the addition of recombinant IL-2, peaking at day 1. GATA-3 showed no upregulation after stimulation with the three cytokines, and its expression was even found to be suppressed after day 3. IL-10 reached its peak expression point at day 1 after stimulation with IL-2 and IL-6, and at day 3 after stimulation with IFN-γ. IL-17A peaked on day 3 in CD4-1^+^ T cells after addition of all the three cytokines, whereas in CD4-2^+^ T cells, only the IL-2 and IL-6 groups peaked on day 3, whereas the IFN-γ group peaked on day 1. IL-6 responded significantly only to IFN-γ and IL-2, peaking on days 1 and 3, respectively. TNF-α responded strongly to IFN-γ and IL-6 stimulation, peaking on day 1 with upregulation.

## Discussion

T-bet plays an important role in the differentiation of naive CD4^+^ T cells into Th1 cells by regulating the secretion of IFN-γ and involved in the regulation of innate and adaptive immunity (13). To date, T-bet has been cloned and characterized only in five fish species (5, 16, 24–26). In the present study, we have analyzed the sequence of T-bet in flounder and found that it has a highly conserved T-box DNA binding domain from fish to mammals. However, flounder T-bet has a limited amino acid identity to mammalian T-bets (43-44%) because of the divergent N and C terminal regions. No signal peptide was found in the structure profile of flounder T-bet molecule, indicating that it was not secreted through the classical pathway and will remain cytosolic. T-bet is a member of the Tbr1 subfamily, which also includes Eomes and Tbr1 (33). Phylogenetic analysis showed that T-bet, Eomes, and Tbr1 form the separate clusters, respectively. In T-bet cluster, mammalian and fish form the separate clusters, respectively. The phylogenetic tree results support that fish T-bet molecules are indeed orthologs of mammalian T-bet.

The high expression of T-bet in the spleen, head kidney, and peripheral blood leukocytes in healthy flounder was observed, which was in agreement with the results from the other fish (16, 25). This result was consistent with the selective expression of T-bet gene in the lymphoid system in mammals (13). Moderate T-bet transcript levels were detected in heart, gill, and brain of flounder, which were in accordance with the previous reports on expression of T-bet in Atlantic salmon (16). The heart and gill are the main organs of blood circulation in fish, some leukocytes with high expression of T-bet may be present. Studies in mice have shown that T-bet plays an important role in the clearance of attenuated rabies viruses (RABV) from central nervous system (34), and we have also found that the expression of T-bet in the brain, its precise roles in the brain need to be further investigated in flounder. In mammals, we know that Th1 cells play a major role in resistance to intracellular pathogens. Therefore, two kinds of intracellular pathogens, *Edwardsiella tarda* and HIRRV, and an extracellular bacterium, *Vibrio anguillarum*, were selected to infect healthy flounder, then the expression of T-bet in the spleen and head kidney was compared. Early and strong T-bet expression was detected in the spleen after the infection of *Edwardsiella tarda* and HIRRV, suggesting that T-bet is involved in the immune response of Th1-type immunity in flounder. Furthermore, T-bet expression in the spleen was also upregulated after *Vibrio anguillarum* infection, suggesting that Th1-type immunity also involved in resistance to extracellular pathogen. Similarly, in the head kidney, *Vibrio anguillarum*, and *Edwardsiella tarda* were able to upregulate T-bet expression, and surprisingly, a moderate T-bet expression was observed after challenge with HIRRV. It can be speculated that no significant immune response after HIRRV infection occurred in the head kidney, whereas the spleen was found to be the major infection target of HIRRV in our previous study (35).

In mammals, T-bet was expressed not only in Th1 cells but also in several different cell lineages of the hematopoietic system, including CD8^+^ T cells, B cells, NK cells, and dendritic cells (16). In ginbuna crucian carp, T-bet was found to be strongly expressed in IgM^-^ lymphocytes at the transcriptional level (25). However, studies on identification of fish T-bet^+^ cells at the cellular level are still lacking, and it is unclear which specific cellular types T-bet is expressed. Therefore, the recombinant protein of T-bet was generated in flounder, and the polyclonal antibody against recombinant protein of T-bet was developed. The antibody specifically recognized the recombinant protein or native T-bet molecule from lymphocytes, and it was able to be used for identification of T-bet^+^ cells in flounder. T-bet^+^ cells were found in the peripheral blood, spleen, and head kidney lymphocytes of flounder, and they were most abundant in the spleen lymphocytes, which was consistent with the result of gene expression in spleen. To our knowledge, this is the first time that T-bet^+^ cells have been identified at the cellular level in fish. More importantly, T-bet was found to be expressed in the nucleus of lymphocytes in flounder. Forkhead box protein 3 (Foxp3), a crucial transcription regulator in the generation and function of CD4^+^CD25^+^ regulatory T cells (Treg), was also reported to be localized in the nucleus of peripheral blood mononuclear cells in tilapia (*Oreochromis niloticus*) (36). Similarly, human T-bet-specific mAb 4B10 could recognize an approximately 62 kDa protein present nuclear extracts prepared from Th1 cell clones, but not Th2 clones (13). These results show that transcription factors act in nucleus to control chromatin and transcription by recognizing specific DNA sequences and forming a complex system that guides expression of the genome (37).

As important immune cells, T/B lymphocytes play an irreplaceable role in cellular and humoral immunity in fish. In our previous study, the monoclonal antibodies against flounder IgM, CD4-1, CD4-2, and polyclonal antibodies against flounder CD8β molecule were produced, respectively, T and B lymphocytes were identified and characterized in flounder (29–31). In this study, T-bet was found to be expressed in sorted IgM^+^ B lymphocytes, and also in CD4-1^+^, CD4-2^+^, and CD8^+^ T lymphocytes. In ginbuna crucian carp, expression of T-bet was also observed in IgM^+^ lymphocytes, although at low levels compared with that in IgM^-^ lymphocytes (25). In mammals, T-bet in B cells was required for IgG class switching and antibody production (17–19), and also found to be upregulated in B cells associated with aging (20). Furthermore, T-bet can respond to viral infection in memory B cells and modulates germinal center polarization and antibody affinity maturation in B cells (21, 22, 38). The class switch recombination is not required in the IgH isotypes in fish (25), and further study on T-bet in teleost B cells is still needed. It was not surprising that T-bet was expressed in flounder CD4^+^ T lymphocyte subsets, because T-bet has been identified as a specific transcription factor of Th1 cells. However, we found teleost T-bet expressed in CD8^+^ T lymphocytes for the first time. In collaboration with another T-box family member, Eomes, T-bet controls the differentiation and maturation of CD8^+^ T cells in mammals (15, 39). The function of fish T-bet in CD8^+^ T cells has not been reported. In addition, T-bet was also expressed in dendritic cells and NK cells in mammals and controls the production of IFN-γ in dendritic cells (40), suppressed the immature gene signature during murine NK cell development (41). Whether T-bet is expressed in dendritic cells or NK cells in fish needs to be further investigated.

Cytokines communicate proximally and distantly *via* autocrine, paracrine, and endocrine, forming a complex system of actions on immune cells that play important roles in physiological and pathological processes, such as immune regulation, inflammatory response, and tumor metastasis (42). In our previous study, the results suggested that IL-6 (43), IL-2 (44), and IFN-γ (unpublished) of flounder could be served as a promising candidate adjuvant and have a potential application in the control of fish diseases. Therefore, to investigate the effect of cytokines on the immune response of flounder T/B lymphocytes and on CD4^+^ T cell differentiation, the recombinant IFN-γ, IL-2, and IL-6 were added to lymphocytes. The percentages of T-bet^+^ cells and T/B lymphocytes, as well as the expression of T-bet and related cytokines in CD4^+^ T cells, were analyzed. The results revealed that the proportion of T-bet^+^ cells appeared to increase after stimulation with all the three cytokines, especially after stimulation with IFN-γ and IL-2, two kinds of cytokines that have been proven to be involved in Th1-type immune responses in mammals. It was reported that T-bet directly transactivates the IFN-γ gene in CD4^+^ T cells and increases the expression of IL-12 receptor β chain on activated cells. Indeed, a positive feedback loop is observed, because STAT1 downstream of the IFN-γ receptor activates T-bet expression, which further serves to increase IFN-γ secretion (45). This positive feedback loop is critical for the proliferation and differentiation of Th1 cells, which are important in host defense against intracellular pathogens. Thus, following IFN-γ stimulation, proliferation of flounder T-bet^+^ cells was found. At the same time, T-bet was mainly expressed in CD4^+^ helper T cells, which explains the proliferation of CD4-1^+^ and CD4-2^+^ T cells in flounder after IFN-γ stimulation. In our previous study, we found that IL-2 was able to induce proliferation of CD4^+^ T cells through specific binding to its receptor IL-2R, and T-bet gene expression was found to be upregulated after stimulation with IL-2 (32), which is consistent with the results of the present study, where IL-2 was able to induce an increase in T-bet^+^ cells and CD4^+^ T cell subpopulations. In the previously study, CD4-2^+^ T cells, rather than CD4-1^+^ T cells have a main role in Th1 immune responses, collaborating with CD8α and CD8β positive cells in flounder (46). And in the present study, a similar pattern could also be found, where a dramatic proliferation of CD8^+^ T cells was observed in IFN-γ and IL-2 stimulated lymphocytes in flounder. In mammalian Th1-type immune response, activated CD8^+^ T cells function as cytotoxic T cells; therefore, it is speculated that CD8^+^ T cells in fish are also activated in the Th1-type immune response (23). The proportion of IgM^+^ B cells showed a trend of increasing followed by decreasing after IFN-γ and IL-2 stimulation, and suspect that some IgM^+^ B cells transformed into plasma cells, which do not express membrane-bound IgM on the cell surface (47). IL-6 is a cytokine that has been documented to be involved in many biological functions, including induce production of acute proteins; participated in host defense against pathogenic infestation and inflammatory responses; induced B cell proliferation, differentiation, and secretion of Ig; and regulated T cell differentiation (48–50). In the present study, T-bet^+^ cells and T lymphocyte subpopulations were not significantly proliferated, whereas IgM^+^ B lymphocytes give a strong response to IL-6, this result consistent with previous studies that fish IL-6 promotes IgM gene upregulation and antibody secretion (51, 52).

In mammals, after antigen stimulation, CD4^+^ cells can differentiate into different subpopulations, and these Th cell subsets play critical and various roles in the innate and adaptive immune system. However, very few functional studies have been carried out on fish CD4^+^ cells due to a lack of tools. Recently, monoclonal antibodies against CD4 molecules were generated for ginbuna crucian carp, rainbow trout, and flounder (31, 53, 54). CD4^+^ cells have been identified using monoclonal antibodies, and the function of teleost CD4^+^ cells was demonstrated to be similar to that of mammalian Th cells. In this study, the transcription factors and cytokines were found to be expressed in sorted flounder CD4-1^+^ and CD4-2^+^ T cells, and the variations of cytokines and transcription factors were largely consistent in CD4-1^+^ and CD4-2^+^ T cells after stimulation. T-bet appeared to be upregulated after stimulation with all three cytokines, especially after IFN-γ and IL-2 stimulation. Similarly, both IFN-γ and IL-2 genes in CD4^+^ T cells formed a positive feedback loop that was significantly upregulated after the addition of recombinant IFN-γ and IL-2, respectively. This would suggest that the differentiation of CD4^+^ Th1 cells also exists in flounder. GATA-3, a Th2 cell-specific transcription factor, is suppressed upon stimulation with the Th1-type cytokines IFN-γ and IL-2, which has the same immune response as in mammals. Indeed, it was reported that T-bet represses Th2 lineage commitment through tyrosine kinase-mediated interaction between T-bet and GATA-3, which interferes with the binding of GATA-3 to its target DNA, and Th2 programs were repressed (55). In addition, IL-10, IL-17A, IL-6, and TNF-α showed different levels of upregulated expression upon stimulation, indicating the complexity and plasticity of CD4^+^ T cell differentiation, and also the extensive and complex functions of cytokine networks in different types of immune responses (2, 3, 56). In conclusion, further studies are needed on T-cell differentiation in fish, and identification of a precise type of Th cell response will shed light on ways to design an effective vaccination strategy against a particular disease (24).

## Data Availability Statement

The original contributions presented in the study are included in the article/supplementary material. Further inquiries can be directed to the corresponding author.

## Ethics Statement

The animal study was reviewed and approved by Instructional Animal Care and Use Committee of the Ocean University of China.

## Author Contributions

HT and JX contributed to the conception and design of this study, performed most of experiments and statistical analysis, drafted and revised the manuscript. XT, HC, and XS participated in the design of the study, helped analyzed experiments and data. WZ designed the study, edited the manuscript, and provided reagents and experiment space. All authors contributed to the article and approved the submitted version.

## Funding

This study was supported by the National Key Research and Development Program of China (2018YFD0900503), Shandong Provincial Natural Science Foundation (ZR2020KC025), the Fundamental Research Funds for the Central Universities (202061020; 201822015), the National Natural Science Foundation of China (31730101; 31672684; 31672685), the Director Foundation of Functional Laboratory for Marine Fisheries Science and Food Production Processes, the Qingdao National Laboratory for Marine Science and Technology (2018MFSD-01), NBRPC (2012CB114406), the Key Research and Development Program of Shandong Province (2016GNC115001), and the OUC-AU joint projects (861901153077).

## Conflict of Interest

The authors declare that the research was conducted in the absence of any commercial or financial relationships that could be construed as a potential conflict of interest.
